# A prospective, observational cohort study to elicit adverse effects of antiretroviral agents in a remote resource-restricted tribal population of Chhattisgarh

**DOI:** 10.4103/0253-7613.58512

**Published:** 2009-10

**Authors:** Harminder Singh, Navin Dulhani, Pawan Tiwari, Prabhakar Singh, Tiku Sinha

**Affiliations:** Department of Pharmacology, Medicine, Anatomy and PSM, Government Medical College, Jagdalpur, Chhattisgarh - 494001, India; 1 Department of Pharmacology, SS Medical College, Rewa, MP, India

**Keywords:** Highly affective antiretroviral therapy, antiretroviral therapy, adverse drug reaction, antitubercular treatment

## Abstract

**Objective:**

To assess the adverse effects of antiretroviral therapy (HAART) and its adherence in HIV-infected patients, in remote and tribal area with restricted resources.

**Materials and Methods:**

This was a prospective, observational study carried out at Department of Medicine, Government Medical College, Jagdalpur. A set of questions were asked and adverse drug reactions (ADRs) were recorded for every patient.

**Results:**

79 HIV positive patients were analyzed. Among them, 68 (86%) had at least one ADR. The mean ADR per patient was 1.64 (±1.09). The most common ADR in our study was peripheral neuropathy (20.83%), followed by skin rashes (15.83%). Twenty-one patients (26.58%) had severe (grade-3 and grade-4) ADRs. Female patients had more ADRs (45.71%) than males (11.36%); severe ADRs had a statistically significant positive correlation with sex and CD4 cell count of the patients.

**Conclusion:**

In spite of high ADRs, HAART is the only answer to HIV/AIDS; thus, management requires a highly precise balance between benefits of durable HIV suppression and the risks of drug toxicity to achieve the therapeutic goals, with conventional drugs or with newer less toxic agents.

## Introduction

Universal access to antiretroviral therapy (ART) is the aim of World Health Organization and UNAIDS.[1] In June 1996, the World AIDS Conference in Vancouver reported new AIDS cocktails and thus ‘highly active antiretroviral therapy’ (HAART) became popular. The potent antiretroviral therapy resulted in marked reduction in the rates of illness and death in the developed world and has led to the effective management of HIV-1 infection.[2] A successful ART regimen involves a combination of at least three drugs. Reduction in morbidity and mortality have been confirmed in all settings in which it has been used, including developing countries.[3] The choice of regimen depends on their side-effect, potential drug interactions, co-morbidities (e.g. tuberculosis, hepatitis), and alternative options in the setting of treatment failure and drug availability and cost.[3] The major individual toxicities include bone marrow suppression (zidovudine), pancreatitis (didanosine), hypersensitivity (abacavir), hepatic necrosis (nevirapine), neuropsychiatric complaints (efavirenz), and nephrolithiasis (indinavir). Hyperlipidemia has emerged as an important concern with HAART, due to the potential for premature atherosclerosis and coronary artery disease.[4] Patients who report significant side effects are more often non-adherent to therapy, 25% of them stop therapy within the first year on HAART and the same number stop because of their concern regarding side effects.[5–7] There are several factors predisposing to antiretroviral-associated adverse events, e.g. female sex, concomitant medications with overlapping and additive toxicities, co-infection with hepatitis B or C, drug-drug interactions, and alcoholism.[8] The present work was carried out to assess the ADR profile of HAART in a remote, tribal area with restricted resources.

## Materials and Methods

This was a prospective, observational study conducted at the Department of Medicine of Government Medical College and associated Maharani Hospital, Jagdalpur Chhattisgarh, with an assistance from the Department of Pharmacology after approval from Institutional Human Research Ethics committee and written consent of patients. The study was conducted from December 2006 to November 2008, in a cohort of 79 patients. All adult HIV patients on HAART therapy of either sex were included. Patients taking antitubercular treatment (ATT) and other drugs with a potential for hepatic or renal toxicity or interactions with HAART drugs and those with a history of alcohol abuse and noncompliance were excluded from the study.

A baseline interview was taken before initiating the therapy followed by further interrogation at first, fourth, and seventh month for the development of ADRs. Information on adverse reactions and data related to health care utilization variables were collected from the medical charts as well as through self-reporting by patients. An adverse reaction to ART was defined as any undesirable effect or symptom registered in the medical charts by the treating physician, that occurred up to 1 year of the first ART prescription.

Baseline laboratory investigations such as hemoglobin (Hb), total and differential leukocyte counts, erythrocyte sedimentation rate, urine analysis, serum venereal disease research laboratory (VDRL) test, serum hepatitis B surface antigen (HBsAg), Mantoux test (MT), and fine needle aspiration cytology (FNAC) of lymph nodes were carried out in each patient to rule out any opportunistic infection or specific contraindications to any drug. The chest X-ray and ultrasonography of the abdomen were done to determine the focus of tuberculosis. Patients were also subjected to liver function tests (LFTs); renal function tests (RFTs), lipid profile, and blood sugar analysis. Clinical examination along with appropriate lab investigations, histopathology, and radiodiagnosis were documented in the adverse reactions reported by the treating physician.

Three types of HAART regimens were used: A: Stavudine, lamivudine, and nevirapine (63%); B: Lamivudine, zidovudine, and nevirapine (30.37%) and C: Stavudine, lamivudine, and efavirenz (6.32%). Allotment of HAART regimens was based on physician's judgment. Data were analyzed using the chi-square test for establishing correlation between severe ADR and different variables. A *P* value < 0.05 was considered to be statistically significant.

## Results

A total of 137 HIV patients were enrolled. Among them, 15 expired, 16 patients stopped the therapy midway, 9 were lost to follow up, and 18 patients were on antitubercular treatment (ATT) simultaneously and so were excluded from the study. Of the 79 patients included in the study, 44 were males and 35 were females. The study group had a mean age of 31.16 ± 8.39 years.

At least one ADR was seen in 68 (86%) patients. The mean ADR per patient was 1.64 (±1.09). The most common ADR observed was peripheral neuropathy (20.83%) and second most common was skin rash (15.83%) [Table 1]. A total of 120 ADRs were noted in 68 patients. Among these 68 patients, 21 had severe (grade-3 and grade-4) ADRs, whereas 47 patients had mild to moderate ADR (Grade-1 and Grade-2). A highly significant correlation between sex and severe ADR was found.

**Table 1:** Number of adverse drug reactions reported to antiretroviral agents (n = 120)

*Adverse effects*	*Frequency (%)*
Peripheral neuropathy	25 (20.83)
Skin rash	19 (15.83)
Lipodystrophy	19 (15.83)
Anemia	16 (13.33)
Abdominal disorders	14 (11.67)
Hepatotoxicity	09 (7.5)
Hyperlipidemia	08 (6.67)
Hair loss	05 (4.16)
Psychological changes	03 (2.5)
Insomnia	02 (1.67)

The incidence of severe adverse reactions was 32.55% in patients less than 35 years of age, as compared to only 19.44% in those more than that. This was statistically insignificantly [Table 2].

**Table 2:** Co-relation of severe ADRs with sex, CD4 cell count, and age of patients (n = 79)

*Variables*	*Number of patients*	*Severe ADR (%)*	*P-value*
Age (in years)			
<35	43	14 (32.55)	0.806
>35	36	07 (19.44)	
Sex			
Male	44	05 (11.36)	<0.001
Female	35	16 (45.71)	
CD4 count (cells/mm^3^)			
<200	33	16 (48.48)	<0.002
>200	46	05 (10.86)	
Regimen			
A	50	11 (22)	0.458
B	24	08 (33)	
C	05	02 (40)	

Among 79 patients, 33 had a CD4 count <200 cells/mm^3^. Sixteen of these patients (48.48%) had severe side effects and in 46 patients who had CD4 count of >200 cells/mm^3^, only 5 (10.86%) had severe side effects which is statistically highly significant. The co-relation between different regimens and severity of ADRs reported were also as statistically insignificant.

## Discussion

In the present study, 79 patients on HAART regimens were observed over a period of 2 years. Adverse effects were seen in 86% of cases. Sharma *et al*. (2008) reported ADRs in 71.1% of cases, and the most common ADR reported was cutaneous manifestation.[9] In our study, the most common ADR reported was peripheral neuropathy (31.64%), mainly with stavudine (d4t) containing regimens. Another study by Kumarasamy *et al*. (2002) has also shown peripheral neuropathy, anemia, and nail hyperpigmentation as the most common side effects. In our study, lipodystrophy was observed in 20.04% of cases, while in other studies it was seen in 14.5% of the cases.[9,10] Saint-Marc *et al*. (2002) reported the incidence of lipodystrophy to be 46.5%.[11]

A study by Malangu *et al*. (2008) reported ADRs in 94% of cases, the most common being a sexual disorder (40%),[12] but in our study, peripheral neuropathy was the most common ADR. In addition, we found that abdominal disorders (11.67%) were the ADRs that affected patient compliance and adherence to prescribed drug regimens.

The correlation between female sex and severe ADR was found to be statistically highly significant. It is not clearly known why sex difference exists in adverse reactions to antiretroviral drugs. Factors cited include differences in weight and body mass index, hormonal changes unique to females, and the effect of these changes on drug metabolism.[13] Other possible factors include differences in fat composition (thereby affecting drug distribution) and genomic differences influencing the level of enzymes involved in drug metabolism.[14]

ADR to ART may depend on the baseline CD4+ cell count at initiation of therapy. A study by Center for Disease Control and Prevention on HIV outpatients suggested that some complications were more frequent and severe when therapy is started at lower CD4+ cell counts. Thus, patients may actually experience fewer side effects over a 10-20 year period of drug exposure if they start therapy 18-24 months earlier than if they delay therapy until the CD4+ cell count decreases to less than 0.200 × 10^9^ cells/L.[15]

The measurement of viral loads and CD4 cell counts and the standard of care for assessing efficacy and response to HAART are not possible everywhere, particularly in resource-restricted places. Also, as eradication of the disease is currently not possible, significant problems related to compliance and long-term toxicity can be anticipated with decade-long therapies. Patient compliance can be improved with proper education and counseling regarding the disease process and inherent but innocuous side effects of HAART. More research is needed to develop low-cost investigations and algorithms for prediction of adverse effects of existing regimen, along with generation of more efficacious and less toxic drugs.
